# Uncovering paradoxes of compassion at work: a dyadic study of compassionate leader behavior

**DOI:** 10.3389/fpsyg.2023.1112644

**Published:** 2023-10-31

**Authors:** Vinzenz Krause, Célia Rousset, Björn Schäfer

**Affiliations:** ^1^Academy for Exponential Change GmbH, Munich, Germany; ^2^Ingolstadt School of Management, Catholic University of Eichstätt-Ingolstadt, Ingolstadt, Bavaria, Germany

**Keywords:** compassion, compassionate leadership, compassionate leader, compassionate behavior, suffering, social hierarchy, dyadic study, compassion at work

## Abstract

In today’s business world, organizations tend to overlook that employees face suffering caused by work and non-work-related events that can negatively impact business organizations in the long run. One way to address this challenge is through leadership acknowledging and alleviating employees’ suffering to ensure a company’s success. However, research on compassion and leadership in business settings is still relatively scarce. In this study, we aim to extend the organizational compassion literature by addressing our research question: “*What are paradoxes induced by compassionate leader behavior in the workplace in the context of social hierarchy?*”. We conducted a qualitative exploratory study based on 12 semi-structured interviews with six dyads of leaders and their direct subordinates from small, medium, and large firms representing different industries. The findings of our study indicate that compassionate leader behavior goes hand-in-hand with paradoxical situations that both leader and member face in the workplace, supporting the proposition that compassion as a social, interpersonal process is complex and multi-faceted. Our analysis identified 6 compassion paradoxes that spring from compassion from a leader towards a member. Our study differentiates from other research of compassion paradoxes in the sense that it also focuses on the interplay between leader and member. From that perspective, the findings of our study indicate that social hierarchy is playing a crucial role and exacerbating some paradoxical tensions. This consideration implies that to be effective, compassionate leaders need to have or develop the ability to continuously transcend those compassion paradoxes, as well as support their members in transcending the paradoxes they find themselves dealing with. Thus, the findings of our study contribute to management literature in the field of Positive Organizational Scholarship (POS) by highlighting compassion as a critical element of dyadic leader-subordinate relationships that could be reinforced by systematically building more competence in leaders and members to navigate the tensions emerging from the identified compassion paradoxes. Additionally, we provide limitations and recommendations for further research, along with several theoretical and practical implications of the results, which are particularly relevant for practitioners such as managing directors, leaders, employees, human resource managers, academics, and business and HR consultants.

## 1. Introduction

Human suffering, both within and outside organizations, seems inevitable. Defined as “the severe or protracted distress people experience when an instance of pain or injury (emotional, physical, or otherwise) disrupts one’s basic personhood” (Kanov, 2021, p.86), suffering springs from many sources. It can come from illness, injury, or even the death of loved ones (Harvey, 2001), from toxic interactions with line managers, colleagues, or customers (Frost, 2003), and also from organizational processes (Maitlis and Ozcelik, 2004) or even from carrying out the “necessary evils” of work organizations (Molinsky and Margolis, 2005). This list of potential sources of suffering shows how pain (and subsequently suffering) is an unavoidable human experience. These psychologically painful experiences can be very costly, not only for the individual who is suffering, but also for the organizations who employ them. In 2003, the Grief Recovery Institute conducted a study showing that companies lose more than $75 billion annually in lost productivity, lost business, and poor performance due to grief-inducing experiences (James et al., 2003). Furthermore, companies today are exposed to tremendous pressure, such as the challenges associated with the COVID-19 pandemic, and are forced to react rapidly to such externalities (Oruh et al., 2021). In contrast to short-notice incidents, organizations are also going through profound transformations through medium- and long-term perspectives in the hope of remaining relevant within their markets. These transformations also create a considerable amount of “pain” in organizations, which then face even more significant challenges to keep their employees engaged and committed (Elias, 2009). Hence, suffering and its associated economic impact will continue to rise in the upcoming years. More than 20 years ago, Frost (1999) stressed that leaders are more and more challenged to deal with the suffering of subordinates, regardless of industry and company size. Today, this remains more true than ever.

During the last decades, researchers have identified different constructs dealing with pain and suffering (e.g., empathy or empathetic response, emotional social support, and prosocial behavior) (Bacharach et al., 1996; Lim and DeSteno, 2016). One such construct is *Compassion* —from the Latin *compati*, meaning “to suffer with.” Even though there are many different definitions of compassion within the organizational research context, a vast and steadily growing number of organizational scholars have drawn on the definition of compassion provided by Dutton et al. (2014). Within their model of the “Interpersonal Process of Compassion,” they define compassion as a human experience comprising of the following key elements: (1) noticing suffering, (2) feeling empathetic concern, (3) sensemaking, and (4) acting to alleviate the suffering (Kanov et al., 2004; Dutton et al., 2014). We also utilize Dutton’s definition of interpersonal compassion, as it allows us to differentiate compassion from other constructs (such as empathy, emotional social support, or prosocial behavior).

While studying compassion is relatively new to the field of organizational behavior, several studies show that compassion at work makes a difference. Frost et al. (2000) demonstrated that compassionate behavior can boost people’s ability to function as productive employees. Further scholars found evidence that compassion also increases attachment and commitment to the organization (Grant et al., 2008; Lilius et al., 2008). Dutton et al. (2002) show that compassionate behavior can strengthen emotional connections at work, and Lilius et al. (2008) outline that it can call up positive emotions and reduce anxiety. Especially in demanding transformation processes, the potential benefits of compassion highlight an alternative for organizations and their leaders in dealing with the increasing amount of suffering, as “compassion offers a potential solution for creating healthier organizations” (Shuck et al., 2019, p. 558). Lilius et al. (2012) conducted a literature review on compassion in the field of Positive Organizational Scholarship (POS) and summarized the benefits of organizational compassion, the processes supporting compassion (Dutton et al., 2002, 2006), the organizational conditions for compassion (Kanov et al., 2004), and the mechanisms of support such as compassionate policies, routines, and systems (Dutton et al., 2002, 2007; Kanov et al., 2004; Frost et al., 2006; Lilius et al., 2008).

Additionally, multiple studies have explicitly investigated the role of leaders within the context of compassion and have shown that leaders are highly relevant in legitimizing the influence of compassion in organizations (see for example Worline and Dutton, 2017). Zoghbi-Manrique-de-Lara and Viera-Armas (2019) found that ethical leadership was significantly and positively linked to compassion and peer-focused citizenship, suggesting that leaders who act morally more easily move their members to become sensitized to their peers’ suffering. Other researchers have called to integrate compassion into previous leadership models, such as servant leadership, as a skill to respond to suffering (Davenport, 2015). More specifically, initial research in the field of compassionate leadership provided the first evidence that compassion does matter in leadership and that it impacts psychological well-being, employee engagement, and turnover intent (Shuck et al., 2019).

However, despite the growing interest in other fields, research on compassionate leadership can still be considered rather scarce and needs further investigation from multiple perspectives. The current state of literature in the field of compassionate leadership and its limited empirical research is summarized best by Shuck et al. (2019) as follows:

“The specific function of compassion related to leader behavior in a work setting remains an underexamined topic across the HRD field as well as in related fields, such as management and organization development. (…) Beyond a limited number of pioneering articles, very little work can empirically comment on how compassion – a behavior that a leader might model – could influence performance within any setting” (Shuck et al., 2019, p. 538).

## 2. Context and objective of our study

Recently, the definition of workplace compassion and the merely positive connotation of compassion has been criticized (Simpson et al., 2014a,b). Former definitions posited that compassion is expressed by leaders towards their members and is described essentially from the perspective of the (powerful) giver and less from the (grateful) receiver (Kanov et al., 2004; Frost et al., 2006; Dutton et al., 2007; Lilius et al., 2008). Simpson et al. (2014a) underline this observation by pointing out that “in the dominant definition the subjectivity of the giver is privileged over the experience of the receiver” (Simpson et al., 2014a, p. 354). Those definitions neglect sociological and political dynamics since they are framed from a concrete psychological position in a unidirectional manner and hence limit the range of the compassion phenomena due to the greater importance of the power relationship of the giver compared to the receiver.

In addition to the rather unidirectional framing of compassion, the previous definitions did not regard compassion as a socially constructed phenomenon intertwined with inherent power dynamics, where those involved may encounter both favorable and unfavorable consequences. On the contrary, many scholars investigating the effects of workplace compassion take an absolutist view on compassion as a virtuous and exclusively positively coined construct (Simpson et al., 2014a,b). In this context, Caza and Carroll (2012) claim that in the “large majority of POS article […] the positive phenomenon was described as inherently valuable, but also having the happy side effect of enhancing profits” (p. 973). From a genealogical perspective, Simpson et al. (2014a) argue that within the social and organizational context, power/knowledge relations and, therefore, analytical, rational, and calculative actions are unavoidable since contemporary leaders tend an organizational arena, wherein “employees may be vicariously treated as the floc – whose obedience is to be cultivated compassionately” (Simpson et al., 2014a, p. 355). Overall power dynamics and adverse outcomes in compassion relations are often underdeveloped and underresearched (e.g., Frost, 1999; Frost et al., 2000, 2006; Kanov et al., 2004; Dutton et al., 2006; Lilius et al., 2011, 2012). The experiences of both leaders and members in compassion relations will likely be multifaceted, complex, continuous, and open to varied interpretations. Consequently, researchers call for more research to investigate organizational compassion through the lens of power dynamics and effects, taking the perspective of both leader and members, and considering the question of who benefits from what knowledge.

Therefore, we are responding to multiple calls for more research in the domain of compassionate leadership behavior by analyzing the complex interplay between leader and member. In this context, we follow the argumentation of previous research (see examples above) outlining that organizational compassion is rather multifaceted and ambiguous in implication and does not only have positive connotations. With our study, we aim to show that organizational compassion is also affected by social hierarchy and may lead to paradoxical situations. We refer to social hierarchy as the differences in power and status among organizational actors (Bunderson and Reagans, 2011). In this context, we analyze the dyadic relationship between leader and member, including both perspectives. Drawing on the work of Araújo et al. (2019) and Simpson et al. (2022), we also presume that the combination of compassion and leadership results in paradoxical challenges (Simpson and Berti, 2020; Tomkins, 2020), evoking the paradoxical poles of certain elements in the compassion context. Organizational scholars describe paradoxes as persistent mutually interdependent but contradictory tensions (Smith and Lewis, 2011). Exploring compassionate leadership through a paradox lens while taking into consideration both the perspective of leader and member constitutes an important contribution as the compassion literature has shown so far limited (empirical) investigation about the tensions experienced by compassionate leaders and their receivers. Doing so could lead to a better understanding of how compassionate leadership works in practice, both for developing theory and for identifying practical guidelines for leaders who wish to bring compassion into their leadership practice. Motivated by that, we state our research question as follows:

*What are paradoxes induced by compassionate leader behavior in the workplace in the context of social hierarchy?*

## 3. Materials and methods

### 3.1. Overview and research sample

The initial study underlying this article set out to deepen our understanding of the effects of compassionate behavior in leader-member settings towards business outcomes in a dyadic study. Thus, we conducted 12 semi-structured, interrogative, dyad interviews with a sample of employees from different companies of different sizes and different industries. To this extent, our sample can be described as a convenience sample from a diverse background of four, only partially overlapping large professional networks (Etikan, 2016).

To identify dyads of leaders and direct subordinates in business organizations, we followed a purposive sampling approach to filter our sample (Palinkas et al., 2015; Bell et al., 2022). We defined a “leader” as taking managerial responsibility for at least one employee. We considered leaders and subordinates from different industries and small, medium, and large-sized firms to cover the relevant phenomena as broadly as possible, thereby ensuring heterogeneity in the sample. We required leaders to have at least 1 year of work experience to maximize in-depth insight into their role as leaders and compassionate behavior. We also required the focal dyad to exist for at least 1 year to allow for a relationship to develop, as compassion can also be related to how well people know each other (Gilbert, 2015). Subsequently, subordinates also needed at least 1 year of work experience to share sufficient practical experience.

Based on these criteria, we contacted leaders and subordinates from 26 companies in the researchers’ networks throughout Germany. Seven contacts gave no feedback and five declined to participate due to time constraints. Two were unwilling to participate as they did not fulfill the above-described criteria and ascribed their reluctance to a perceived weak relationship and insufficient exchange that would make difficult to answer any dyadic questions. In total, 12 contacts agreed to in-depth interviews. The final sample is, thus, composed of 12 participants from six dyads consisting of four women and eight men aged between 22 and 59. To ensure anonymity and openness, we conducted the interviews of each individual of each dyad separately. Four dyads stem from large enterprises, one dyad stems from a medium-sized enterprise, and one dyad from a small-sized company, representing six different sectors. The relationship duration of the dyads ranges from 1 to 3 years. The members’ (M) professional experience ranges from one to 14 years, while the leaders’ (L) management experience ranges from 1.5 to 16 years. The participants work in different areas such as International Marketing (M01; L01), Equities (M02; L02), Sales and Distribution (M03; L03), Project Management and Video Production (M04; L04), UX Design (M05; L05), and Marketing Communication (M06; L06). Due to the COVID-19 pandemic, all interviews were conducted virtually *via* Zoom and Microsoft Teams, with activated cameras. The average duration of the interviews conducted in German was 43:00 min.

### 3.2. Interview guidelines

Our initial study aimed at studying the phenomenon of compassionate leadership in business organizations in a broader, open-ended way through the investigation of the effects of compassion in leadership, the individual reasons for compassionate behavior, and the limits and challenges of compassion in leadership in the organizational context, while providing deeper insights into the dyads’ dynamics. Therefore, we based the questions of our semi-structured interview guideline on the existing compassion literature and LMX theory (Liden et al., 1997). The team prepared the guide according to the rules of Lindgren et al. (2020), emphasizing an open-but-targeted form of questioning (Miles et al., 2020). Due to the study setup, we developed separate guides for leaders’ and members’ interviews. They included statements concerning confidentiality between the dyad members, prohibiting the researchers from sharing insights from previous interviews with the second part of the dyad to promote openness even in sensitive cases (Eisikovits and Koren, 2010). The guidelines were tested thoroughly in advance through two test interviews with both a leader and a member and were subsequently slightly adapted (Miles et al., 2020). The final interview guides consisted of an introduction, a main part comprising 12 questions for leaders and 11 for subordinates, and a conclusion. In addition to an icebreaker question, the first block of questions aimed at introducing the topic. After asking participants how they would define compassion, we also provided a working definition to ensure a common understanding during the interview. For simplicity, we used an updated definition from Gilbert, who defines compassion as a “sensitivity to suffering in self and others with a commitment to try to alleviate and prevent it” (Gilbert, 2014). The second block of questions sought to explore the status quo of compassion in leadership within the participants’ business organizations. The questions strived to identify the general attitude toward compassion and cover all aspects of the compassion process, especially expressing, noticing, and responding to suffering. The last block addressed drivers and barriers to compassionate behavior in leaders.

### 3.3. Data analysis

The interviews were recorded, fully transcribed, and initially underwent a structured deductive content analysis. While the initial intent of our study was to deepen our understanding of the effects of compassionate behavior in leader-member settings towards business outcomes in a dyadic study, we narrowed our focus during the data analysis and review process leading to this article. In our preliminary analyses, two insights came to light: (a) the relevance of social hierarchy-relations and (b) the paradoxical situations which can arise in the context of compassionate behavior in organizations. In line with iterative approaches in qualitative analysis (see Srivastava and Hopwood, 2009; Miles et al., 2020), we decided to follow those insights and shifted towards inductive content analysis. From that point on, our qualitative analysis was conducted interactively with comparisons between the data and the literature (Corbin and Strauss, 2008). We first started by scanning our interviews for evidence of paradoxical tensions as defined by Smith and Lewis (2011). In particular, we were looking for evidence of poles that “seem logical in isolation, but absurd and irrational when appearing simultaneously” (Lewis, 2000). In this endeavor, we also made sure to distinguish paradox from dilemmas, which require a difficult choice between competing alternatives that each have advantages and disadvantages, but that can be resolved temporarily by integrating contradictory elements (Clegg and Cunha, 2017). Dilemmas become paradoxical only when options are contradictory and interrelated such that any choice between them is temporary and tension will resurface (Smith and Lewis, 2011). After a first run through the data to reveal paradoxical tensions, we then went back to the data and conducted the first level of coding by analyzing the data to identify and code core themes with the intention to identify discrete types of paradox. This analysis generated six main categories. Going back and forth between data and literature, we compared our findings with the literature to identify and classify similarities and differences (Eisenhardt, 1989; Eisenhardt and Graebner, 2007).

The overall data analysis was run by two researchers independently who, in case of unclarity or conflicting categorization of information between both researchers, consulted a third researcher who acted as a neutral “judge” to ensure a high level of objectivity. In addition to that, within the data analysis process, all coders were also screening for examples of compassion which leader and member (dyad) referred to similarly. This allowed us to directly compare (1) the behavior of leader and member in a given situation, (2) their perception of provided and received compassion, as well as (3) the shared views or expectations in the exact same situation. Our dyadic research approach therefore enabled us to analyze the two sides of most situations which were described during the interviews.

## 4. Results

*“[…] suffering is anything that describes a deviation from the norm that pulls me down personally and/or negatively affects me, in every respect. Suffering among my employees can be manifold. The classic is suffering at work caused by the job, but also suffering at work can be caused by a private situation, and suffering can be caused by a private situation. […] But I think I have less to do with the latter now, because there is relatively little I can do about it. What I can control are all the sufferings that ultimately condition the work or influence the work”* (L06).

As this leader eloquently shares, suffering can spring from many sources, and impact – or not – the workplace. This reality is creating a tension between what belongs to the private and what belongs to the professional life. Indeed, our interviewees often pointed out the line between private and professional being blurred:

*“if something is really completely in the personal space, you might want to separate it[…]. That could include if there’s anything going on with my girlfriend. That’s something I would tend to keep private. But I think the line is rather a bit blurred. So, if my grandmother were to die now […] that would certainly also be emotional, personal and intimate suffering. And I would probably share that anyway, at least to the extent that I want to communicate it so that others can better classify my situation – and thus also with my supervisor”* (M02).

Not surprisingly, in our sample, our interviewees raised the point that the boundary varies from one person to the next. One leader for example states that some people *“are also very conservative and do not really want to reveal anything about themselves”* (L02), while others state very clearly that compassionate leadership means for them that they are also able and allowed to share private information (M03).

As stated by L06, people will bring suffering from home to work (but also vice versa), and the line between private and professional becomes blurred. According to our analysis, expressing and responding to this suffering in the workplace leads to six paradoxical tensions (see Table 1). Interestingly, even though we did not deliberately look for paradoxical tensions for each step of the Interpersonal Compassion Process, 5 out of those 6 can be mapped to that process, while the last one relates to the leader-member relationship. The Table 1 provides an overview of those categories, and how they map to this process.

**Table 1:** Analysis categories: identified paradoxes mapped to the interpersonal compassion process.

Interpersonal process of compassion (Dutton et al., 2014)	Paradoxes identified in our study
A sufferer’s experienced and expressed suffering	Paradox of showing the vulnerable, private self versus showing the strong, professional self
A focal actor noticing suffering	Paradox of probing (pro-)actively but respecting boundaries
A focal actor feeling empathic concern	Paradox of compassion requiring empathy and understanding, yet leaders need to show discernment so as not to be exploited or manipulated
A focal actor and sufferer engaging in sensemaking	
A focal actor acting compassionately	Paradox of the imperative for compassion to answer one person’s suffering versus the imperative for equality and fairness to other team members
	Paradox of weakness versus courage
*Non applicable*	Paradox of the impact on the leader- member relationship: distance (hierarchy) versus closeness (interpersonal bonds)

In the following sub-sections, we will present those paradoxical tensions and illustrate them with quotes from our interviews. Each quote is labeled to a particular interviewee, with “M” indicating a “member,” “L” indicating a “leader,” and the subsequent number representing the corresponding dyad. Finally, we will also present our findings relevant to another paradox of strategic versus selfless compassion that Araújo et al. (2019) identified.

### 4.1. Paradox #1: showing the vulnerable private self versus showing the strong professional self

When suffering, the employees we interviewed often mentioned a paradoxical dilemma they face when deciding if they should express their suffering or not, especially in the context of the hierarchical relationship with their supervisor. One of them explains:

*“it’s always a bit of a balancing act: To what extent do I show myself vulnerable or somehow sensitive, weak? To what extent do I allow a person who is superior to me and who, if necessary, can and may also make decisions about my further professional future, to have insight into how I am doing emotionally or what any of my problems are. Of course, this is always a matter of consideration”* (M04).

The desire to show a strong professional self is linked to the concept of impression management, defined as the process by which people control the impressions others form of them (Leary and Kowalski, 1990). On the other hand, showing vulnerability implies deliberately disclosing sensible, potentially damaging information (Nienaber et al., 2015). In the context of suffering and compassion, while followers expect a certain level of compassionate leadership behavior, they need to first express that suffering, requiring them to show their vulnerable private self and (potentially) eroding their strong professional self, which they think they need to retain their jobs and not create additional suffering (immediately or in the future).

One of the leaders interviewed displayed a good understanding of how vulnerable employees can feel when sharing their suffering:

*“And if the other person smashes you or shows no understanding, then I imagine it like a snail that just gets out of the shell and at that moment pulls itself back into the house very, very quickly, and then getting out again is relatively difficult” (L06).*

The need to control the impression others form of them led some of the members interviewed to carefully consider which suffering they would share or not to not be perceived too weak for a current or prospective job:

*“If there are any psychological problems now, I would perhaps also be cautious, because regardless of the corporate culture, I believe psychological suffering can also be taken up as excessive demands and also weakness and as a lack of qualifications for the job. I’d probably really hold back on that. […] All suffering that goes in the direction of being overwhelmed at work and also being overwhelmed by a lack of qualifications - I would consider whether I share it that way”* (M03).

*“At the moment when one reveals private problems or personal problems that may now have to do not only with work processes, but with personal feelings or circumstances, one naturally makes oneself vulnerable, which of course in the case of […] a promotion […], this can make you look weaker than someone else possibly” (M04).*

Our interviewees also point out the risk that being vulnerable and sharing their suffering might impact how performance is reviewed – positively or negatively, consciously or unconsciously:

*“When it comes to performance reviews, a leader with compassion and with different relationships with employees no longer evaluates 100% objectively” (M03).*

*“To be honest, I believe that if something like this happens too often, that at some point you can also assess the person a little less. So, I think it can change perception” (M05).*

One leader also recognizes that performance evaluation can be a challenging exercise, indicating that it can be difficult to compartmentalize and disregard some sensitive topics they are aware of and that are affecting their employees’ performance.

*“You have to differentiate very strongly between the classic performance that you have to evaluate as a supervisor and you have to differentiate that from possible compassion, problems or whatever kind of thing you have knowledge of. Because you are responsible for evaluating performance purely from the job description. Quite simply, it becomes clear when an employee is sick and can only work three days a week. Then I’m only allowed to evaluate these three days and I’m not allowed to evaluate the 5 days where I say, “He′s not there for 2 days.” And that’s ultimately how it is with the topic. If I am aware of a topic that has been openly brought to my attention, then I must hide it in the classic evaluation, but I have to evaluate the pure work performance. This is sometimes difficult” (L03).*

Interestingly, our interviews indicated that the coin can be flipped, and strength can be experienced when sharing vulnerability.

*“And that was a very concrete example, where the feedback came that this person had been thinking about this vulnerability or this supposed weakness to talk, but then to make it known, and that then helped the person extremely to draw strength, because she did not have to hide it, but was able to deal with it openly towards me” (L03).*

In addition, the experience of compassion has been indicated by an employee as a way to go past their fear of being vulnerable, thereby creating a virtuous circle:

*“I think through the compassionate behavior of my leader, I am encouraged to communicate better and share more. And because of that, there are fewer situations where you are just dissatisfied, but you do not change anything about it, because you do not even get to share it because you think: “Well, I’d rather not say anything, because that could be used against me or be interpreted negatively” (M04).*

### 4.2. Paradox #2: probing actively while respecting (individual and unclear) boundaries

*“To what extent can I impose myself as a managing director and as a boss? […] How far can I get involved or try to penetrate the emotional world of the other person? How far am I allowed to do this? I think you must hold back and think carefully about when you can hook in and when you cannot. And ultimately, I believe that compassionate leadership is right at the moment when you turn the offer into a conversation. And if that is accepted, then you can devote yourself to it. But if that is not accepted, the offer, then it is actually none of my business, that is a private story” (L04).*

Most of the employees we interviewed insisted on the fact that they expect their leaders to ask and probe when they notice their suffering, especially as sometimes it can be easier for them to continue a conversation about their suffering initiated by their leader rather than start one. Also, they expect their leaders to observe and intervene if necessary:

*“And then, if necessary, to ask back. Or at some point, for example, in a different professional context, when my boss also notices that things were not going well or maybe he noticed that I was somehow different than usual. Then maybe ask “what is it?”” (M01).*

This perception is mostly aligned with the leaders we interviewed, but they place an equally important responsibility on the employee to share (or not), as illustrated by those leaders:

*“[Being] compassionate is to perceive [suffering], that’s the first thing. The fact that you perceive: “Okay, something seems to be wrong.” and then also as a manager to have an ear there, sometimes to ask a question, just to listen […]. And then it is also up to the respective employee himself to decide how deeply and what he actually gives of himself” (L03).*

*“And you also make a comment: “Ah, you are not in such a good mood today.” And then you usually observe: What is the reaction of the person? And either the one goes into the conversation […] or he does not say anything. But I’m not one to drill now. So, people have to reveal themselves” (L01).*

The example above hints at the concern to invade too much the space of their members (or their “emotional world” as stated by L04 as the start of this sub-section). Because the nature and experience of suffering is so individual, most of the leaders we interviewed tend to take a more cautionary approach and prefer to leave it to their employee’s initiative to initiate a conversation. As illustrated in the example below, in some cases leaders can also feel a tension between listening and asking for more details:

*“There are limits where employees are more likely to want to keep the private private and he also kept private for a while. But only when it became so big and had so much influence on professional life, only then has he communicated it to his supervisor, although we also have this buddy relationship. Nevertheless, he wanted to keep it more private. I think that also shows that there are definitely limits and I respected that to a certain extent, that he does not want to talk too much about it and I have not asked for every single detail – there is definitely a limit” (L02).*

One leader in particular also feared that if they would probe too much – even if guided by the desire to help their employee – they might be perceived as abusive in their position of hierarchical superior:

*“If I become abusive and interfere in very special, private problems or create pressure or ask: “Now come, now tell me about it, I notice it very clearly.” […] The danger is definitely there that you get too close to people. So not as a leader, but that you just bring the aura into the aura that everyone considers their privacy. I think indiscreet behavior is multi-layered, annoying and also disrespectful and as such it can also be understood as a lack of respect or as arrogance” (L04).*

To manage this tension between the expectation to notice the suffering and ask about it without invading the privacy of their members, one leader uses his value of respect as a North Star: *“[You] need to have a clear respect for the individual – when someone signals that they do not want to go one step further, to accept that” (L03).*

### 4.3. Paradox #3: empathetic and understanding versus discerning

*In general, […] if you are always helping, you are fostering a little bit of a “me-me-me” mentality and like this: “Oh, […] If I feel like it, then I’ll tell him how bad I’m doing and then he’ll give it to someone else.” There’s a fine line, I think – depending on how well you can work with your people – between “I’ll help you because you need it” and “I’ll let you take advantage of me because you do not feel like doing anything” or generally just say: “Man, I’ll tell you how bad my weekend was and with my dead grandma he’ll somehow cover my projects for me this week.” […] That’s why I think the chance of being exploited is immense (L06).*

As this leader pointed out, our interviews revealed that some leaders feel a clear tension between being and showing understanding towards their members who need help, while applying enough discernment to avoid being exploited by those who might be tempted to take advantage of a leader’s compassionate attitude. This paradoxical tension is also reflected in our interviews with the members who express their clear expectation toward leaders to be empathetic and understanding, while recognizing that same risk of exploitation and manipulation.

Indeed, on one hand, employees in our sample expect their leaders to take them seriously and feel their suffering. As a matter of fact, all members interviewed define compassionate leadership as their leader showing a deep understanding and empathy for their issues and suffering. They expect their leaders to put themselves in their shoes and show a genuine interest for them, sometimes even to show forgiveness for a temporary dip in motivation or performance. Even when there is no obvious solution to solve their problem or alleviate their suffering, the minimum they want is to be heard and seen:

*“What I would always expect is at least to get the feeling of being taken seriously. There does not always have to be a solution to everything, but I want to have the feeling that I can express my problems without her saying “But that’s not so bad,” or “just pull yourself together” […]. Because at the moment when I see this as a problem and go so far as to communicate it, I expect my counterpart to take it seriously. As I said, there is not always an immediate solution for everything, or possibilities to implement this as one would like to wish or hope for. But I always expect that at least this will be taken seriously” (M04).*

This member goes further and shares an example when they felt they were not taken seriously when they shared that they were overwhelmed with the amount of work they had:

*“I was actually flattened that it was dismissed that way and that’s this certain situation, where in retrospect a lot of things come up and in the second you are so surprised. It’s also unpleasant to admit something like that you cannot do it anymore. But the fact that it is then made so small, so to speak, and really wiped away, I was really overwhelmed to address it further, because I was simply irritated, I have to admit. […] So, you can always wipe away a lot of such problems. That’s the feeling I had rather. In this respect, I did not have the impression that he had really acknowledged this” (M04).*

At the same time, while employees want to be taken seriously, they also recognize that their leaders’ compassion could be exploited:

*“It can also be exploited if a leader is too compassionate. If people say: “I’m not feeling so well today” and the manager says: “Yes, then you close the computer and lie down” – of course, this can also be exploited if someone says: “I do not feel like working today. I’ll say I’m sick,” or something. […] It can also be that there are people in the team who say: “Oh, our boss, he’s very compassionate. I’m sure he’ll understand if I have to visit my mother now because I have not seen her for 2 years since the lockdown.” And in the middle of the most stressful phase of the campaign (…) So I think this exploitation is an issue” (M06).*

### 4.4. Paradox #4: imperative for compassion towards one sufferer versus the imperative for equality and fairness toward the rest of the team

Within their Paradoxical Leadership Behavior (PLB) framework, Zhang et al. (2015) define the paradox for leaders of “treating subordinates uniformly while allowing individualizations.” This paradox refers to the challenge for leaders of providing their members with “identical privileges, rights, and status without displaying favoritism” (p. 542), while ensuring members are not depersonalized or deprived of their unique identity. In the frame of compassion, actions to alleviate suffering might create a temporary situation where members benefit from different privileges and rights. When addressing the suffering of one of their team members, the leaders we interviewed pointed out the difficulties in answering the needs of this person in a tailored manner and alleviating them while ensuring equality and fairness with the rest of the team. On one hand, providing an individualized response was often perceived by our interviewees as necessary to alleviate the suffering of the sufferer. On the other hand, they also defined equality as being fairly treated, not seeing favoritism behaviors, and as everybody having the equal chance to receive compassion from their leaders. This paradox also resonates with 2 organizational compassion paradoxes proposed by Simpson and Berti (2020): *Accept unfairness* versus *promotes fairness*, and *unjust* versus *just*. For the former, the scholars base their paradox on the research of Thompson (2007) and Du Gay (2008) who argue that compassionate administration is an arbitrary and unfair expression of favoritism. For the latter, they base their paradox on the research by Batson et al. (1995) indicating that compassion leads to decisions that conflict with justice.

Some of our interviewees recognize the risk of perceived favoritism, which can create tensions within a team:

*“This can also have the effect of saying, “The boss prefers someone.” You feel unfairly treated. Of course, this can be the case – especially when a new employee comes along and sees: “They already have a long-standing working relationship. Would I get so much sympathy from the boss if I said my dog was sick now?”” (M06).*

*“It’s also a bit of a question: How much compassion do you show to whom? So as soon as you do not show everyone the same amount of compassion, because you may like some better than others – happens as a leader sometimes – then of course you quickly have such a favorite that people say: “Man, […] she’s his darling anyway, she can do whatever she wants; cough, then you can stay at home for 2 weeks. And then it’s all up to us again” (L06).*

Some recognize that the quality of the relationship between a leader and a member might influence the level of compassion displayed:

*“The question is: are all employees shown the same amount of compassion? This is not always the case. Some more than others, depending on how far the personal relationship between the supervisor and the employee is. […] So, inequality arise and also the feeling that in the worst case some people do not take advantage of it now, but they constantly need this compassion, so to speak, because something is constantly not working, where you can ask yourself: Why is it always the case with you that something in your life does not work, and then you always have to bring it with you to work?” (M05).*

In some cases, when leaders have to deal with confidential information, it can be even more difficult to ensure that the rest of the team does not see any favor treatment:

*“There was a person who had a case of cancer in the family […] and not everyone in the team should know. And of course, the team should not think that the person is now favored when he or she may not have to perform or do quite as much as the others” (L05).*

In other cases, a respondent suggested that the inequality might simply arise from the fact that people would tend to describe their suffering differently, thereby leading to different solutions to address that suffering:

*“The disadvantage may be that employees feel treated unequally with each other with the solutions that are presented […] which may actually depend on how the respective person describes their problem or possibly even comes up with suggestions for solutions [themselves]. But that there might be an imbalance and people feel disadvantaged” (M04).*

Even when the rest of the team is fully behind the actions of the leader to alleviate the suffering of another team member, it can create a feeling of inequality, especially as often the rest of the team needs to work harder to support that person. A leader comments:

*“On the other hand, the number of projects is not decreasing. But if you have two hands less, you have to ask your other people to do more – and of course beyond their regular working hours – and to do so with pleasure” (L06).*

Another interesting micro-discourse from a team member goes in a similar direction, highlighting the contradictory feelings that this paradox can evoke, starting with envy towards the sufferer followed by guilt as a secondary emotion, while suffering themselves from the consequences of the solutions and the perceived unfairness:

*“I know a colleague who has gone through some crisis every week, with his relationships and family, and which I also find quite terrible. But that was really a big burden for the team. I know how the conversations took place between my boss and the person. But for a short time, I thought to myself […]: We would all like to have so much freedom from time to time, just not to work, not to finish a project after all, because you just get more time. And that was also at the expense of other employees, who then had to finish it on top, or who always had to argue why things are not there, which is not always pleasant. So once is not once, but from the fifth, sixth time it just gets difficult at some point” (M05).*

In this case, the member goes further in describing the felt unfairness due to the fact that the attention of their leader was only directed to a few people and who consequently felt left aside:

*“I was just unlucky. Well, I think so, maybe I had just insinuated that I get along well with everyone anyway. That’s why I do not have any problems at all. And other colleagues who were not as powerful were more likely to get this help because they thought: “Oh, they are not quite as stable,” so you have to look at them. […] So, it felt very unfair to me because every employee was now given very personal advice, but I had the feeling that there were just a few that were overlooked. Some because you say: “Oh, they are not that important anyway.” Some where you say: “Yes, they can do it on their own anyway.” (..) I did not think that was fair or balanced” (M05).*

Interestingly, the corresponding leader in this situation seemed oblivious to the fact that some of his team members felt overlooked in this situation:

*“Of course, I do not differentiate in such a way that it appears that I prefer someone. Rather, the employees then see that everyone has the same amount of attention, communication, or time together in the one-on-ones. Or we have time together. So, you should definitely not have favorite employees” (L05).*

According to our interviews, this tension is exacerbated by the fact that suffering is very individual and subjective. Therefore by default, not everybody would have the same threshold, or feel the same amount of suffering in comparable situations.

*“Everyone defines suffering differently for themselves. One of them may have grown up with the hamster and the other says: “Yes okay, my grandma died, but I’m not going to act like that now, I’m pulling myself together now.” It’s totally different, but at that moment both are equally bad for the respective person” (M06).*

This also led the leaders to doing some sensemaking to try and preserve equality in the team, and to accommodate for the fact that different employees might communicate differently and suffer differently:

*“And there is always so much talk about Generation Z, that they are so demanding. And if they do not like something, they complain right away. And they are so idealistic. I think you have to differentiate again, whether employees sometimes go through stressful phases, they should also consciously. And some things they just have to swallow. That’s how I would look at it” (L02).*

To resolve that tension, some explain that it is important to refocus the group and take a perspective:

*“And it cannot be that someone thinks: “Okay, he has advantages” by showing compassion and being a little closer to the person, that the others think I prefer him. So, this must not happen, but it must remain in a context where everyone sees: Okay, it is now about the situation and not about the compassion in the context of a favor, but to help the person in the moment” (L01).*

### 4.5. Paradox #5: compassion leader behavior as a sign of weakness or requiring courage

Sadly, leaders in our interviews shared that in their organization their compassion is often perceived as weakness by top management, which can sometimes even stand in the way of career advancement.

*“I have more of a problem in the other direction that I’m too soft. My superiors tell me: “You’ll only become a real manager once you fire your first employee. […] there is definitely a risk that […] I will not be seen as a hard manager who can also enforce things” (L02).*

This tension with upper management is also quite clear for some members, who describe the challenges leaders face when they act in a compassionate manner:

*“It comes across as weakness for some – not perhaps for the people in the team, but just one level higher – if compassion is shown, that it may be counted as a sign of weakness […]. I’ve experienced that too. [My supervisor] is very compassionate. In the upper tiers it is already thin. There is often little understanding or […] no proactive questioning: “How are your teams doing?” And in case of doubt, you are also quickly there with termination agreements” (M06).*

Those results align with the “*Sign of weakness* versus *Requires courage*” paradox that Simpson and Berti (2020) identify as part of their proposed organizational compassion paradoxes. They base this proposal on the research of Koerner (2014) stating that “for the giver of compassion in organizational settings, courage is typically associated with relational power imbalances, manifesting in actions that threaten relationships with more powerful individuals” (p. 444).

This lack of alignment on values with the organization can create a lot of frustrations for leaders who are trying to alleviate the suffering of their employees out of altruism but also with the intention to maintain the performance level of the organization. Sometimes, the frustration is made even bigger by the fact that the organization is sending a message feigning compassion but that, in action, leaders are often left alone.

*“In general, an employer does not make it easy for you to release an employee, to create freedom for him – for this, generally large companies are not created. They do not really want flexibility, but actually a large company wants to know at all times where the employee is, what he is doing and why he is doing it. The more you demand from the employer […] the more difficult it will be in the end. You often get very, very frustrated – and that would be the second emotion – because you hit a wall again or it did not go any further. […]*. *You feign a lot of compassion, you have that in your corporate targets, you have it anchored in your values […] But I do not think most big companies live that. And you realize relatively quickly, as soon as you want to take advantage of a value like this and need help, the fun is over relatively quickly. It’s always about performance, business, topics, goals, KPIs – that’s upheld. Most companies are compassionate as long as it does not affect their KPIs. I do not think any company would put an employee above its own corporate goals” (L06).*

Other leaders agree that it is unrealistic that a company would put an employee above its corporate goals and call for a balanced approach in order to “understand the goals of the employee when there is a conflict of goals, and try to resolve it in communication, that you bring both company goals and employee goals into harmony “(L05). To solve that tension, some of the leaders we interviewed are calling to transcend that paradox and really bring compassion as a core value in organizations.

*“I am also a company representative and have to make sure that the performance of the company is as it should be; that the pace we need is maintained and that the topics that are relevant to us in business are driven forward. The more suffering my employees have and the more aches and pains, the less they will be able to maintain this pace, the less they will be able to maintain the standard, the less the quality will correspond to what everyone imagines. That’s why compassion must also play a role from a company’s point of view, otherwise you burn people and you do not get your topics on the street. And then, you can see it in KPIs and sales figures. Then you shoot yourself in the foot. In the short term this may work, but in the long term/medium term, I do not think it works” (L06).*

### 4.6. Paradox #6: impact on leader–member relationship: distance (hierarchy) versus closeness (interpersonal connection)

The blurred line between professional and private life induced by suffering and compassion in the workplace also impacted the relationship between the leaders and members we interviewed. The experience of compassion tended to bring leaders and members “closer,” leading them to question how close is appropriate. Zhang et al. (2015) identified a similar paradox in their conceptualization of paradoxical leadership and argue that leaders are challenged by the need to maintain distance – through vertical structural relationships and differentiation in status, rank, authority and powers – while simultaneously forming interpersonal bonds (and therefore minimizing status distinctions).

One leader interviewed recognized clearly that addressing the suffering of employees in the workplace and taking an interest in their emotions and distress can create a tension for other leaders who would rather remain in the role of the distant and aloof superior:

*“[Showing compassion to employees brings] flatter hierarchies automatically. […] I also know other bosses who are very concerned about hierarchies. They would certainly also perceive that they consciously build up a distance and do not want to hear the personal issues not to let this closeness arise since it comes with a responsibility that you may have to take on, but this is additionally burdensome” (L04).*

Even the leaders we interviewed who were convinced by the benefits of compassion at work recognized that there were some risks in getting too close to their members, including a risk of losing respect or authority.

*“When a problem arises, […] you also give the employees the space to become abusive towards me and then the necessary respect – not because of my age or position, but simply because of the company structure and the fact that there are always decision-makers, you take very much out of me and sometimes cross a border” (L04).*

*“The limit is really reached when I no longer have authority and the employees no longer do what I want. But at the end, the employee has to do what I want. He is welcome to give me input and share his opinion, but if I then say: “No, we’ll do it this way now,” then that has to happen. And I think if I do not have the authority anymore that he does not do it, that would be a limit where it becomes difficult” (L02).*

Going beyond the concept of Zhang et al. (2015), our findings also outlined the experience of the member. Interestingly, some members also identified clear risks associated with a close relationship with their leaders, including the fact that it can make difficult conversations even harder to conduct.

*“Everything that has to do with my direct job and also with success is absolutely appropriate to be interested in it as a boss. And showing interest in personal matters is great, also appropriate, but there you must not forget: You still have to keep your professional distance. You cannot think of your colleagues or your boss as your closest friend. Of course, it can happen that you build it up outside the office, but in the end it’s still about the success of the company. And if you build up a personal relationship, they might be setbacks. And my boss once gave an example that he had to decide on a person he had to dismiss. He also told me that he was sleepless in bed because it was extremely difficult” (M03).*

Another interesting micro-discourse illustrates well the tightrope members walk between the necessary distance and desired closeness with their leader, and how it could impact the work result:

*“At some point you fall too far out of this professional context. When you realize you have a great relationship of trust there, that you may no longer have only through this professional relationship, but at some point it will go into your private life in a certain form […] and then I could imagine that the boundaries will become blurred […]. My boss is totally empathetic, helpful, courteous – yes, these are actually exactly the qualities I expect from this compassion – then I think to myself: “Oh yes, actually we get along well and should meet sometimes..” Then maybe there are some discrepancies […]. And then, of course, it reflects one-to-one on the professional relationship and I think then you can often misinterpret topics, or interpret too much into them. And of course, this can also worsen a work result” (M01).*

They add on, commenting on how closeness can bring confusion in terms of hierarchy levels, as well as difficulties to engage in difficult conversations such as salary negotiations.

*“I think then it would just be too much of a friendly relationship for me. And in a friendly relationship, I think, you are still on one level, but in a professional situation, my manager is of course superior to me. I think, it’s just difficult – also from a communication perspective – that you still maintain this. He (leader) is actually above you, but actually behaves like me on one level, but there are also various topics where this superordinate and subordinate is absolutely relevant. For example, when I’m talking about salary negotiations and I just find it difficult, if you are too much on this friendly basis […]. I think, it’s very difficult to discuss with your management, at a serious, constructive level” (M01).*

### 4.7. Strategic versus selfless compassion: a theoretical paradox?

Within their research, Araújo et al. (2019) emphasized paradoxical tensions between compassionate motives that are selfless and strategic. They define selfless compassion as springing from internal ethical virtues or conscience, whereas strategic compassion describes the value or organizational compassion as a contributor to enhanced organizational performance, productivity, and profitability. Within our study, we also found evidence for those two motivations existing simultaneously in the leaders we interviewed. Indeed, on one hand leaders mentioned that they are driven by ideals of humanistic values, personal determination, and prior experience in one’s own personal and professional environment regarding suffering. Acts of selflessness appeared especially in situations where suffering occurred in a very personal context of the member, such as the illness of children, indications of burnout, or very strong and deep relations and commitment to the team/team members. For instance, one of the leaders provides an example:

*“I took away one topic completely [from the member] during the time when the children [of the member] were ill, and I simply said: “Watch out - if anything happens, be it a child suffering, you have to go to the doctor” [..] then I simply jump in. We do a short update beforehand [..] get me on board, I go to meetings, I take over certain activities in the meantime, we do a short handover afterward” (L06).*

This leader also adds that “compassion is important and it’s a given.” Interestingly, his member confirms the statements and the selflessness of his leader by extending the scope of incidents:

*“For example, if the child is sick, then he says: You take care of the child. If I have too much work, then he says: “Give something, tell me what, and I’ll take care of it.” Or […] when he notices that we have been working so late again in the evening, he says: “On Friday, we finish work at two o’clock”” (M06).*

This example shows that the act of selflessness is recognized and is not intentionally expected but appreciated by the member.

At the same time, leaders in our interviews outlined that they also behave compassionately towards their members for strategic reasons, noting an increase in member performance, productivity, motivation, job satisfaction, commitment, and retention to the company. For instance, some leaders stress the link between compassion and performance:

*“Compassion is definitely an issue that is important because it ultimately increases employee performance and satisfaction. [...] I think satisfaction is important for employee retention. Thus, I would always say: show compassion, be empathetic. That increases employee satisfaction and performance. And that’s also measured in our company” (L02).*

*“It’s simply performance enhancement. I really mean it. An employee who feels comfortable, who feels understood, even sometimes in other situations will perform three times better than an employee who does not feel understood” (L03).*

In most of our six dyads, leaders highlight the strategic impetus of their compassionate behavior but often also link it to personal reasons/motivations. For example, one leader explains his compassionate behavior from a strategic and personal perspective:

*“That is multi-layered. I am also quite honest about that. On the one hand, I’m a very pragmatic person: My task is to ensure that work performance is maintained. On the other hand, because I have been through a lot, through a relatively early death of my father, when I was 15 [...]. In my time, a lot has happened in my immediate environment and a lot has happened to me - I have a lot behind me. I think that’s when you become sensitive to something like that” (L04).*

*I want to actively do something about it, because it is important to me personally. [..] I want to change something about it because I care about it for my team and for the success of the company (L06).*

One of the most striking outcomes in our study revealed all six members interviewed are aware their corresponding leaders show compassionate behavior rather due to strategic motives than pure selflessness. This underlines the fact that employees in our study are evaluating the compassionate behavior of their leaders critically and that they are aware of the primarily non-altruistic behavior of their leaders when it comes to alleviating their suffering. For most of the members interviewed, this compassionate leadership behavior is acceptable. Regardless of the motive behind the compassion, what they eventually expect is for their suffering to be noticed, responded to, empathized with, and appraised accordingly both at the workplace and partially in personal terms when it has an impact on the work performance of the member.

*“Of course, you can say that this is simply altruism. At the same time, I’m also sure that this increases team performance because suffering employees are not performant employees. And, of course, the bond with the employer. When I feel in good hands. So, when things get tight, when I know: Okay, my employer will take care of it at this point, is helpful. Then maybe I’ll stay longer with my employer” (M05).*

Other members confirm the strategic intentions of their leaders and attribute it to an organizational and team point of view:

*“AI believe that there are always financial considerations involved because otherwise, an employee who is really in a crisis will effectively leave. [...]. It is perhaps not only a problem for the employee himself [...], but also for the whole team, because an employee who is in a crisis also influences the team” (M05).*

Finally, some members raise the positive effects of their leader’s compassionate behavior for the organization but also for themselves, even if strategic motives cause it:

*“I think that’s also very important in terms of performance. If you feel good, you work more productively. Then it’s also very important when it comes to proactive problem solving: If you show compassion, you also work more transparently, you address problems more readily. In the end, it’s also more beneficial for the company, that’s for sure” (M03).*

In addition, our findings add a different facet to the “strategic” motive of compassion. Indeed, our interviewees revealed that the strategic decision to show compassion can be self-serving. Effectively, beyond increasing organizational performance, some leaders indicated that acting to alleviate the suffering of their employees can be necessary for their own image and reputation in the organization, to ensure their own personal success as manager.

*“Above all, it is usually the case that what others may perceive as suffering is relatively easy for you to solve – in 80 percent of the cases it is just somehow a snap of your fingers, it does not hurt you. And in the end, it always pays off. So, let us put it selfishly: Showing compassion as a boss always pays off in the end […] the topic of loyalty or motivation is of course what you want to buy as a boss” (L06).*

Interestingly, even this self-serving dimension of advancing the leaders’ goals is apparent for some members as well:

*“Ultimately, happy employees are also just effective employees. So, also believe that it is in his (leader) interest if I suffer less, and presumably also work better. Ultimately, he also wants his team to advance his goals” (M01).*

Some members also refer to a feeling of indebtedness towards their leaders that pushes them to do a good job in return of their leader’s compassionate behavior:

*“If he shows compassion, she will also hope that I will be open to him and that I will not come up with any excuses in any form for any topics and then perhaps feel obliged in a different way, because I also know he will also respond to me. Then I also make sure that I do a good job during the time when I’m there” (M06).*

Therefore, our results first indicate that leaders in our study could be driven to behave compassionately (or not) in the workplace due to a leadership strategy to guarantee results. The recognition and full acceptance of this reality by members in our sample indicates a mutual understanding that compassion within the relation between leader and member does not need to follow pure selfless motives to alleviate the prevailing suffering. From our interviews, it seems that leaders need to balance 3 areas: serving up (upper management, company goals), serving others (members, often out of altruistic, humanistic motives), and serving oneself (maintaining position through results, career development), and that those 3 areas might have an influence on the decision to act in a compassionate manner. However, we did not find evidence that any of our interviewees felt a paradoxical tension emerging from those opposing motives behind compassionate behavior. Instead, most of them rather presented those different motives as the different sides of a coin or as different facets of their motivation. They often referred first to their personality and values, giving them a certain sensitivity to others’ suffering and triggering an emotional motivation to act to alleviate that suffering, then they added the more rational and strategic reasons that legitimize a response in the workplace context. Therefore, even though those various motivations might be theoretically paradoxical, the actual presence of a paradoxical tension as defined by Smith and Lewis (2011) is ambiguous in our data. For this reason, we did not include this dimension as a paradox that leaders and members face when leaders demonstrate compassionate behavior in the workplace.

## 5. Discussion

### 5.1. Critical discussion of key findings

#### 5.1.1. Compassion paradoxes induced by compassionate leader behavior

The findings of our study indicate that compassionate leader behavior goes hand-in-hand with paradoxical situations that both leader and member face in the workplace, supporting the proposition that compassion as a social, interpersonal process is complex and multi-faceted. Whereas one could think that compassion is a straightforward act, our study shows that it brings many considerations for both leaders and members.

Our analysis has identified 6 compassion paradoxes that spring from compassion from a leader towards a member. Little research so far has shown interest in the perspective of the receiver of compassion. This has recently been criticized by researchers (see for example Simpson et al., 2014a,b) who called for further attention to the receiver’s experience. In our analysis, 1 out of 6 paradoxes is faced by the member/receiver, 4 out of 6 are faced by the leader/ giver, and 1 is faced by both. To our knowledge, some paradoxes are new and have not yet been explored in literature. Some paradoxes have been recently explored by other researchers and our study builds on them (for example Zhang et al., 2015; Simpson and Berti, 2020). Therefore, our study contributes to a growing body of literature studying the intersection between compassion and paradoxes, while giving a voice to both leaders and members.

#### 5.1.2. The impact of social hierarchy on compassion paradoxes

Hierarchy is a defining and pervasive feature of organizations (Magee and Galinsky, 2008). Our study differentiates from other research of compassion paradoxes in the sense that it also focuses on the interplay between leader and member. From that perspective, the findings of our study indicate that social hierarchy is playing a crucial role and exacerbating some paradoxical tensions. In particular, we argue that social hierarchy plays an important role in 4 out of the 6 paradoxes identified.

##### 5.1.2.1. Showing the vulnerable, private self versus showing the strong professional self when expressing suffering

Our findings have shown that members sometimes face a difficult decision when considering sharing their suffering or not. Indeed, the person who is the most able to offer them a solution to respond to their suffering is also the person who is evaluating their performance and making, or at least facilitating, career decisions. In that sense, members can feel that their leaders have “power-over” them (Göhler, 2013), meaning that they have the capacity to enforce their will over others. This accentuates the fear of being perceived as vulnerable, weak, too demanding, incapable, thereby impacting the decision of expressing suffering. Once compassion has been received, this can also lead members to feel indebted toward their leaders and that they need to work harder to compensate (Clark, 1987, 1998; Clegg, 1989; Schmitt and Clark, 2006; Simpson et al., 2014b). Even though our study has not shown evidence of leaders consciously manipulating their members into such position, research has shown that givers of compassion might unconsciously engender a sense of diminished agency as dependency, obligation, indebtedness, and even emotional enslavement (Szasz, 1994; Stirrat and Henkel, 1997; Simpson and Berti, 2020).

##### 5.1.2.2. The imperative for compassion and an individualized response to suffering versus the imperative for fairness and equality

Providing an individualized response to a suffering member might bring a sense of unfairness (Thompson, 2007; Du Gay, 2008), injustice (Batson et al., 1995), and an overall challenge for leaders to provide all members with “identical privileges, rights, and status without displaying favoritism” (Zhang et al., 2015, p. 542), while ensuring members are not depersonalized or deprived of their unique identity. Indeed, in the frame of compassion, actions to alleviate suffering might create a temporary situation where members benefit from different privileges and rights. In a sense, we could argue that when practicing compassion, leaders might choose equity over equality. The decision for equity, however, might conflict with the expectations of team members. Scholars have hypothesized that organizational culture would determine which distribution principle (equity, equality, or need) group members would use to allocate resources (Mannix et al., 1995). Other scholars have shown that conflicts can stem from equality violations and result in nondirected conflict that is symptomatic of decreased social cohesiveness (Kabanoff, 1991).

##### 5.1.2.3. Compassion as a sign of weakness versus requiring courage

Our findings indicate that leaders find themselves sandwiched between their employees and their own managers, sometimes including different sets of expectations. Although previous research indicates that compassion is a quality of leaders that leads to a higher perceived leadership competency and to being acknowledged as a successful leader (Kellett et al., 2002; Mahsud et al., 2010; Melwani et al., 2012), some of the leaders participating in this study fear the opposite. Indeed, they are themselves embedded in a web of hierarchy and need to constantly watch out to maintain their status in the organization. This status is naturally deeply influenced by the organizational context since compassion is shaped not only by personal and relational contexts, but also through norms and values exhibited at the organizational level (Dutton et al., 2014; Kanov et al., 2017; Yoon, 2017). The attitudes of managers in our interviews are particularly criticized in such contexts, leading to some participants fearing their compassionate behavior might impact their career development. This represents the dimension of courage, which is associated with relational power imbalances, manifesting in actions that threaten relationships with more powerful individuals (Koerner, 2014; Simpson and Berti, 2020). Worse, sometimes the leaders in our sample found themselves stuck between two narratives: the official narrative of the organization who claims to be human-centric and compassionate, and the unofficial narrative that showing compassion and vulnerability equal weakness. This echoes Simpson and Berti (2020) who state that „in social contexts characterized by a prominence of power abuse upheld by systemic power that normalizes social relations as taken for granted, compassionate action can involve challenging existing inequities, placing the giver in a vulnerable position of great risk, and necessitating a great deal of personal courage” (Simpson and Berti, 2020, p. 444).

##### 5.1.2.4. The impact of compassion on the leader-member relationship: distance versus closeness

The results of our study have shown that the fact whether the leader-member relationship is considered and intended to be more distant (focused on hierarchy) or close (focused on interpersonal connection) depends on contextual and personal factors. In this context, the Leader-Member-Exchange theory (LMX) as a developmental and dynamic process focuses on the dyadic relationship quality between leader and follower as a social exchange process (Graen and Uhl-Bien, 1995). According to Graen and Scandura (1987) leaders are developing different working relationships with their members and the relationship quality with the member is influencing the effectiveness of the leader. Studies have shown that high-quality relationships, driven by communication, trust or mutual respect and support, correlate with important outcomes such as job satisfaction, commitment or high work performance (Fairhurst and Chandler, 1989; Liden and Maslyn, 1998). Interestingly, the LMX theory does not provide any insights on how the degree of distance or closeness might impact the quality of the relationship between member and leader (and therefore effectiveness and performance). It could be argued that close relationships are beneficial for both leaders and members. However, the results of our study in the context of “distance versus closeness” have revealed contradictory insights. On the one hand, particular members are looking for establishing close, interpersonal relationships by also sharing private information and granting their leader access to their private life. On the other hand, certain members are clearly distinguishing between the private and professional spheres and therefore define distant and close relationships differently. A similar logic applies for leaders. Additionally, while the participants we interviewed hinted at the fact that compassion brought them closer with their leader or member, some of them recognized some potential difficulties that could stem from this proximity, especially around professional respect, which incidentally is also a LMX dimension. This indicates that compassion at work brings both leaders and members to act in a field of tensions stemming from the maintenance of the hierarchical structure and the partly necessary interplay between the private and professional sphere.

#### 5.1.3. Compassionate leadership as the ability to manage and transcend compassion paradoxes

So far, there is no consensus among researchers about what compassionate leadership truly is. Some argue that compassionate leader behavior is merely a skill of servant leadership (Davenport, 2015). Others have attempted to define its core dimensions that would set it apart from other leadership styles (see Shuck et al., 2019). Worline and Dutton (2017) differentiate between *leading with compassion* (which “entails using a leader’s interpersonal skills to alleviate suffering in work interactions with followers,” p.174) and *leading for compassion* (which “involves becoming a high-level compassion architect,” p.182). The latest research around compassion paradoxes adds a new dimension to what it could mean to be a compassionate leader. Even though paradoxes cannot be resolved, they can be transcended so that an individual or organization can embrace the inherent tensions simultaneously (Smith and Lewis, 2011). Since those tensions are inherent to organizations where “competing demands cannot be resolved but rather continually resurface” (Smith et al., 2017, p. 307), some researchers such as Simpson and Berti encourage us to approach the transcendence of organizational compassion as an ongoing accomplishment. This consideration implies that to be effective, compassionate leaders need to have or develop the ability to continuously transcend those compassion paradoxes, as well as support their members in transcending the paradoxes they find themselves dealing with. This is especially important since, quite ironically, the paradoxes inherent to compassion practices in organizations are sometimes causing some suffering in leaders and members. We would argue that this even creates a deeper responsibility for organizations and their leaders to act towards the facilitation of the transcendence of those paradoxes, since the potential suffering they create might be preventable (Kanov, 2021).

There is no consensus today on how to transcend a paradox. Poole and van de Ven (1989) argued that “it is possible that the paradox may stem from conceptual limitations or flaws in theory or assumptions. To overcome these limitations it is necessary to introduce new concepts or a new perspective” (p. 567). Based on yin-yang philosophy, Zhang et al. (2015) encourage us to see paradoxes as “structurally and individually ambidextrous,” arguing that the two sides “coexist, like yin and yang, depending on and complementing one another to jointly support leader effectiveness in people management” (p.541). More recently, Simpson and Berti (2020) argued for an ongoing model in which “each day, the tensions will present themselves, providing an opportunity to make them salient and deploy cognitive, discursive, and socio-material transcendence strategies to enact wise compassion courageously supported by generative power-to, both systemic and individual.”

We believe that transcending the compassion paradoxes in the workplace by introducing new terms and theory will allow for the humility to accept that those paradoxes will not be solved once and for all but will have to be navigated each day. In turn, this might pave a way towards changing the system and equipping leaders and members systematically to develop their competence further in handling those inevitable tensions that arise from putting the human at the center (Poole and van de Ven, 1989). As we have learned through this study and others, compassion in the workplace is challenging because it is a human, subjective experience. The degree of attention to suffering expected by leaders and members, the expected response, and many other elements vary from one individual to another. Compassion has many faces and cannot be reduced to a box to be ticked, otherwise we would risk for it to be (perceived as) fake or manipulative. However, if we were to develop a new framework that recognizes compassion at work as the new “normal,” including a corresponding structure and processes, and at the same time recognize that leadership today is all about handling paradoxes, we believe that it would allow to develop healthier and more effective practices of compassion in the workplace.

### 5.2. Limitations and future research

This study is based on a qualitative research approach, entailing limitations. Collecting and analyzing our data, we followed a rigorous methodology and considered quality criteria. The sequential dyadic approach reduces to some extent the risk of social desirability bias, as participants are aware that their statements can be cross-referenced. We explicitly granted both anonymity and confidentiality, explaining no information would be shared with the other part of the dyad. Using carefully worded, open questions, we assured participants no answer was wrong, thus increasing reliable output. Our sample was derived from our wider network and not from a general population. During the recruitment process of the participants, it was observed that potential participants who had positive relationships with their professional counterparts were more willing to participate in this study than those with less positive relationships. While our interviews did reveal (intended) negative (side) effects of compassionate behavior in leader-member dyads and overall organizations, dyads with a lower relationship quality could even stress this aspect more prominently.

Moreover, our sample lacks diversity regarding the gender ratio of the leaders, as the dyads are characterized by male leaders only. Since theory indicates that personal characteristics, such as gender, have an impact on compassionate behavior (Goetz et al., 2010; Dutton et al., 2014; Peticca-Harris, 2019; Banker and Bhal, 2020), this may have influenced the findings of the study. According to the interview data, one employee even suggested that organizations dominated by men can have a deleterious effect on compassionate behavior. Although the researchers considered a homogenous distribution in the recruiting process of the participants, the final sample is dominated by men. Therefore, future research should ensure a larger sample size with an equal ratio of male and female leaders. A comparison of males’ and females’ perspectives may provide further interesting insights. Since the sample consists only of German leaders and employees from an individualistic cultural background, it lacks cultural diversity and limits the findings to Western culture. Thus, further research should also consider participants from different cultures to provide a valuable contribution to research. Furthermore, it is also important to note that the interviews were conducted, coded and analyzed in the native language of the interviewees (German).

Due to the small sample size of 12 participants, the results have limited generalizability in the context of business organizations (Malterud et al., 2016). Thus, it seems reasonable to further validate the results of this study by increasing study-size or even turning to a longitudinal study design. Moreover, even though our original interview guideline was not initially designed for covering paradoxes in compassion, future research can build on our results and adapt the interview guideline provided by us accordingly. While our sample was limited in size and to several industries, future research still should consider examining larger samples of leaders and employees from additional industries to investigate the negative (side) effects of compassionate leadership. Further, there is evidence from the interviews that the type of organization, its culture, and its size may be linked to the level of suitability for leader compassion and the influence of power on the relationship. Finally, data collection through the interviews was limited by the current COVID-19 situation and, therefore, could only be conducted *via* video conferences, which could have affected the results. Although the study suggests that working from home due to the pandemic has an impact on compassion between leaders and employees, the scope of this study did not encompass the role of the pandemic. Therefore, this could be an interesting additional aspect for further research.

### 5.3. Implications for theory

This study contributes to the understanding and critical reflection of paradoxical tensions induced by compassion in the workplace by revealing new paradoxes faced by leader and member. It includes both leaders’ and members’ perspectives and addresses the call for further research into how firms can authentically reduce employees’ suffering and promote their well-being (Frost, 1999; Peus, 2011). As previous research mostly involves theoretical studies, this study contributes by following an explorative empirical approach with the focus on dyads, therefore providing insights and perspectives that have not yet been examined by existing management literature. More precisely, we produce initial evidence for new compassion paradoxes inherent to compassion leader behavior at work, as well as confirm and develop on previously identified paradoxes (Araújo et al., 2019; Simpson and Berti, 2020). Drawing upon the previous works of Simpson et al. (2014b), Araújo et al. (2019), and Simpson et al. (2022), we find indications that leaders often show compassion towards their members not only out of selfless reasons but due to strategy and rationality, calculating the resulting organizational effects, such as an increased commitment and willingness to raise efforts or stay with the team. As all of our six dyads revealed such calculations (either existing, perceived, anticipated), this raises the question to what extent” compassionate leadership” can be seen as such and how to delimit it from other forms of leadership. While compassion is more traditionally associated with altruistic motives, future research on organizational compassion might want to acknowledge openly and from the beginning the more strategic aspects motivating compassion behavior in the workplace.

At the same time, several dyads showed the potential of over-utilizing leaders’ compassionate behavior by members, leaving leaders in the paradoxical situation that showing too much (authentic) compassion will lead to more stress-related suffering by other members. Moreover, our study revealed that members also own certain power and therefore might abuse and exploit the compassionate behavior of their leaders to their benefits – and to the detriment of the organization. Future research should explore deeper the dynamics between leader and member, and shed some more light about the power members have over their compassionate leaders. In addition, since our results also showed that power dynamics are prevalent and are being used even in strong dyadic relationships, it would be of high interest to investigate to which extent the issue of power plays an even more crucial and dominant role when researching weak relationships. We also found indications of leaders allegedly showing compassion which, in fact, belittled and patronized their members or even highlighted their deficiencies. The analysis of our dyads provided limited evidence, but some interviewees reported such situations from previous job experiences. While compassionate leadership does yield a lot of positive results, scholars need to control for negatives in empirical studies. Future research thus cannot continue to solely focus on the positive aspects of compassion but has to include a focus on trade-offs and ambiguous or even paradoxical situations. Addressing power issues in particular will be particularly important in order to facilitate the emergence of real compassionate leadership (Simpson et al., 2014b; Simpson and Berti, 2020). Finally, future studies could focus on bringing to life a new model for compassion at work to transcend the current inherent paradoxes.

### 5.4. Implications for practice

Our study raises several issues which leaders and members should consider when giving or receiving compassion at work.

First, the paradoxes induced by compassion at work need to be acknowledged. Pretending they do not exist might create false expectations and ironically, more suffering. Instead, we encourage organizations and their leaders to make these tensions salient and engage in dialogue about them. In this acknowledgement, we would also encourage all participants to challenge the “either – or” mindset towards a “both – and” mindset, where they can see the two sides of a coin, like yin and yang, depending on and complementing one another to jointly support bringing more compassion at work.

Second, leadership development programs, independent of a focus on compassionate leadership, should raise awareness among participants concerning dealing with tensions and partially paradoxical situations when showing compassion. Since one of the most critical aspects for leaders is to deal with extreme emotionality of members in certain situations, these programs should also aim at upskilling leaders to react, respectively, to those situations. Moreover, leadership programs also should address how leaders can avoid manipulative or overreaching behavior, offering or even forcing unrequested compassionate behavior upon their members. Comparably, leaders’ sensitivity to the dangers of being perceived as patronizing or even belittling their members must be raised in such programs. Equipping leaders with the ability to communicate the advantages of compassionate behavior as well as drawbacks for the entire team when misused by the members also should be included to raise the overall awareness for appropriate organizational behavior.

Thirdly, leaders and organizations must be aware of the changing perception of compassion over time, which is not only initiated by organizational members but can underlie public perceptions. Thus, practitioners must frequently reflect upon the appropriateness of compassionate behavior shown at any point to avoid a negative backlash of presumed positive behavior. Even more importantly, organizations and its leaders at all levels should reflect on the risk of advertising a compassionate culture without living it practically.

## 6. Conclusion

We started this paper by highlighting the cost of suffering for individuals and business organizations. We also argued that the amount of suffering in the workplace is likely to continue to increase as companies deal with external and internal challenges in their pursuit of remaining relevant and successful. While compassion has proven to be a natural answer to suffering and has shown to bring many organizational benefits, little is yet known about the role leaders have to play in bringing more compassion to the workplace and how the dynamics between leaders and members in the context of compassion are structured.

Through the analysis of the perceptions and actions of both leaders and members in a dyadic empirical setting, we could reveal novel insights in the context of compassionate leader behavior by identifying six compassion paradoxes faced by leader and members. Our analysis also focused on the impact of social hierarchy in the workplace on compassion paradoxes. Finally, our research findings bring us to argue that finding a way to transcend the compassion paradoxes instead of only balancing them, might help resolve the tensions inherent to those paradoxes more sustainability and build more competence for compassion in the workplace.

Researchers and practitioners can play an important role in transcending these paradoxes and we describe some levers that can be utilized to create an environment where compassion can flourish so that organizations and their people can reap the benefits of alleviating and preventing suffering in the workplace.

## Data availability statement

The raw data supporting the conclusions of this article will be made available by the authors, without undue reservation.

## Author contributions

CR: conceptualization, writing – original draft, data analysis, writing, and governance. VK: conceptualization, writing – original draft, data analysis, writing, and funding acquisition. BS: writing, review, editing, and funding acquisition. All authors designed the research, contributed to the article, and approved the submitted version.
